# The complexity of mating decisions in stalk‐eyed flies

**DOI:** 10.1002/ece3.3225

**Published:** 2017-07-18

**Authors:** Nadine C. Chapman, Penthai Siriwat, James Howie, Aaron Towlson, Lawrence Bellamy, Kevin Fowler, Andrew Pomiankowski

**Affiliations:** ^1^ Department of Genetics, Evolution and Environment University College London London UK; ^2^ School of Life and Environmental Science University of Sydney Sydney NSW Australia; ^3^ CoMPLEX University College London London UK

**Keywords:** courtship, genetic variation, male mate choice, mate preference, multimodal signaling, multiple sexual traits, sexual ornament, sexual selection

## Abstract

All too often, studies of sexual selection focus exclusively on the responses in one sex, on single traits, typically those that are exaggerated and strongly sexually dimorphic. They ignore a range of less obvious traits and behavior, in both sexes, involved in the interactions leading to mate choice. To remedy this imbalance, we analyze a textbook example of sexual selection in the stalk‐eyed fly (*Diasemopsis meigenii*). We studied several traits in a novel, insightful, and efficient experimental design, examining 2,400 male–female pairs in a “round‐robin” array, where each female was tested against multiple males and vice versa. In *D. meigenii*, females exhibit strong mate preference for males with highly exaggerated eyespan, and so we deliberately constrained variation in male eyespan to reveal the importance of other traits. Males performing more precopulatory behavior were more likely to attempt to mate with females and be accepted by them. However, behavior was not a necessary part of courtship, as it was absent from over almost half the interactions. Males with larger reproductive organs (testes and accessory glands) did not make more mating attempts, but there was a strong tendency for females to accept mating attempts from such males. How females detect differences in male reproductive organ size remains unclear. In addition, females with larger eyespan, an indicator of size and fecundity, attracted more mating attempts from males, but this trait did not alter female acceptance. Genetic variation among males had a strong influence on male mating attempts and female acceptance, both via the traits we studied and other unmeasured attributes. These findings demonstrate the importance of assaying multiple traits in males and females, rather than focusing solely on prominent and exaggerated sexually dimorphic traits. The approach allows a more complete understanding of the complex mating decisions made by both males and females.

## 1 INTRODUCTION

Sex is a complex interplay between individuals of the two sexes. Many factors such as quality, experience, and competitive ability influence mating decisions across space and time (e.g., Andersson, 1994; Cotton, Small, & Pomiankowski, 2006; Miller & Svensson, 2014). Despite this complexity, many studies have adopted a simplifying approach of evaluating the importance of variation in individual traits, usually in one sex (typically males). Much attention has been paid to species where males bear traits that are attractive to females or are used in aggressive competition for mating opportunities (Andersson, 1994). Often, these species involve exaggerated male sexual traits (ornaments and weapons) that can be easily quantified and sometimes manipulated by researchers (Andersson, 1994; Bonduriansky, 2007; Kraaijeveld, Kraaijeveld‐Smit, & Komdeur, 2007).

While prominent sexual ornaments (and female preferences for them) and weapons are important, other traits in both sexes that are less obvious, or not so easily measured, are likely to explain a considerable fraction of the variance in mating success. It is often overlooked that males use several criteria to decide whether and to what degree they court females or choose between available females (Bergstrom & Real, 2000; Bonduriansky, 2001; Cotton, Cotton, Small, & Pomiankowski, 2015; Edward & Chapman, 2011; Green & Madjidian, 2011; Hooper & Miller, 2008; Kokko & Johnstone, 2002; Servedio & Lande, 2006). Species with exaggerated sexual ornaments frequently possess a suite of other less extravagant but nonetheless dimorphic morphological traits (“multiple sexual traits”) that are displayed together with several complex courtship behaviors (Candolin, 2003; Girard, Elias, & Kasumovic, 2015; Hebets, Stafstrom, Rodrigues, & Wilgers, 2011; Jones, Byrne, & Wallman, 2014; Lehtonen, Rintakoski, & Lindstrom, 2007; Patricelli, Uy, & Borgia, 2003). Similarly, multiple traits influence fighting ability and contest resolution, not just the main weapon (Hall, McLare, Brooks, & Lailvaux, 2010; Lailvaux, Herrel, Vanhooydonck, Meyers, & Irschick, 2004; Zeng, Zhu, & Kang, 2016). In addition, females are not passive partners but interact with males during courtship, leading males to persist with or abandon mating attempts (Stockley & Bro‐Jorgensen, 2011).

In order to more fully understand the varied mechanisms that underpin mate choice and the resultant selective pressures on both sexes, it is vital to adopt inclusive experimental designs that account for the multiple traits involved in male–female interactions (Girard et al., 2015; Jones et al., 2014; Parton, 2013). These considerations motivated this study of stalk‐eyed flies, model organisms for studies of sexual selection (Chapman, Pomiankowski, & Fowler, 2005). In a number of stalk‐eyed fly species, male eyespan is a highly exaggerated, condition‐dependent trait (Cotton, Fowler, & Pomiankowski, 2004a,b; David et al., 1998; Knell, Fruhauf, & Norris, 1999), subject to directional female preference (Cotton, Rogers, Small, Pomiankowski, & Fowler, 2006; Hingle, Fowler, & Pomiankowski, 2001; Wilkinson & Reillo, 1994) and provides indirect genetic benefits, both for good genes (Bellamy, Chapman, Fowler, & Pomiankowski, 2013; David, Bjorksten, Fowler, & Pomiankowski, 2000) and against meiotic drive genes (Cotton, Földvári, Cotton, & Pomiankowski, 2014; Wilkinson & Reillo, 1994), and direct fertility benefits for females (Harley et al., 2013). Male eyespan also plays a role in intrasexual antagonistic interactions to determine ownership of lek breeding sites, with larger eyespan males being victorious more frequently in confrontations (Burkhardt & de la Motte, 1983; Small, Cotton, Fowler, & Pomiankowski, 2009; Wilkinson & Reillo, 1994).

As in other species influenced by an obvious sexually dimorphic trait, there appear to be several subtle male behaviors involved. For example, in the African stalk‐eyed fly, *Diasemopsis meigenii,* males follow females while bobbing their abdomens (personal observations, Chapman). In addition, females take an active role in rejecting some male mating attempts by extension of their ovipositors to prohibit copulation and vigorous body shaking to dislodge mounted males. In the Malaysian stalk‐eyed fly, *Teleopsis dalmanni*, there is evidence that male reproductive organ size (testes and accessory glands) affects mating rate. Males with larger testes and accessory glands attract more females to their lek sites (even after controlling for body size covariation; Cotton, Small, Hashim, & Pomiankowski, 2010), and these well‐endowed males gain more matings under controlled laboratory conditions (Baker et al., 2003; Fry, 2006; Hingle et al., 2001; Rogers et al., 2005; Rogers, Chapman, Fowler, & Pomiankowski, 2005; Rogers, Denniff, Chapman, Fowler, & Pomiankowski, 2008; Wilkinson, Kahler, & Baker, 1998). In addition, observations in the wild as well as laboratory experiments demonstrate that *T. dalmanni* males neither mate at random nor with all females attracted to their lek sites, but rather exert mate preference for females with larger eyespan and higher fecundity (Cotton et al., 2015).

The aim of this study was to evaluate the importance of a set of additional traits beyond male eyespan. We examined how variation in female eyespan, male reproductive traits (testes and accessory glands), and male behavior directed at females affected male–female interactions. We measured two outcomes, male mating attempts and female acceptance or rejection of those attempts. Mating attempts appear to be largely under male control, as males approach and mount females. In contrast, acceptance or rejection of these mating attempts appears to be largely under female control, given that female action leads to the dislodging of unwanted male mounting. We obtained our focal males from a set of highly inbred lines and an outbred control (Bellamy et al., 2013). This enabled us to determine whether there were male genetic effects on mating attempts and acceptance, and hence, whether there are genetic benefits to females associated with male–female mating interactions. In order to highlight any role of the additional candidate traits, we severely constrained variation in male eyespan. We also removed the opportunity for intrasexual antagonistic interactions (either in males or in females) to influence mate choice, by using an assay design that paired single males with single females.

The experimental work was set up on a scale much larger than is typical of mate choice assays. In part, this was motivated by the expectation that the noncanonical traits we were looking to assess were likely to have less extreme consequences for mating. We analyzed the responses of 240 males, sequentially presented to 10 females, in total requiring observations of 2,400 male–female pairs. In order to make this a feasible approach, male–female pairings were evaluated in a round‐robin design, so that each set of 10 females was provided sequentially with each of 10 males. In this way, an observer typically monitored the mating behavior of 100 pairings per day, gaining information on 10 females and 10 males. This proved to be an efficient, balanced, and sufficiently large‐scale way of obtaining concrete evidence about the mating decisions made by each of the sexes.

## 2 MATERIALS AND METHODS

### 2.1 Source populations

Two populations provided flies for this study. An outbred stock population of *D. meigenii* was founded from flies collected by S. Hilger in 2000 from South Africa. These flies have been maintained in laboratory cage culture (>200 individuals to minimize inbreeding) at 25°C, fed pureed corn twice weekly, on a 12:12 hr light:dark cycle, with fifteen‐minute artificial “dawn” and “dusk” periods (reduced illumination) at the start and end of the light phase.

A suite of inbred lines was also used that had been created by pairing virgin males and females at random and then enforcing brother–sister pair matings for 11 generations (Bellamy et al., 2013). At the end of this period of intense inbreeding (inbreeding coefficient = 0.926, probability of fixation = 0.859; Falconer & Mackay, 1996), lines were established in population cage culture under the same conditions as those of the stock outbred population. These lines constitute snapshots of the genetic variation in the laboratory population. There was no selection imposed during inbreeding beyond that invoked by inbreeding itself, which has the effect of eliminating those lines in which strongly deleterious recessives had been made homozygous. This was evident in that of 105 original lines, 27 survived to become established in population cage culture. As survivors, these lines represent nondeleterious genetic combinations. We drew males from 12 of the surviving lines for the experiments reported here. Males from each line were largely homozygous and, more importantly, genetically distinct from those in other lines. The presence of significant effects due to line is indicative of segregating genetic variation in the morphological, reproductive, and behavioral traits that we monitored. We also compared the inbred line males to outbred stock population males. This was in order to calibrate whether the inbred flies were in deficient in any way in their traits and degree of mating success.

### 2.2 Flies for experiments

Experimental male flies were generated from inbred and outbred line cages by inserting petri dishes lined with moist cotton wool and excess pureed corn and collecting eggs. Eclosed males were separated from females before reaching 3 weeks of age to ensure virginity. At least 48 hr before use, flies were anaesthetized on ice, measured for eyespan (distance between the outermost point of the eyes) and thorax (from the top of the head to the apex of the third set of legs) to the nearest 0.01 mm using NIH ImageJ (Abramoff, Magalhaes, & Ram, 2004; Schneider et al., 2012), and placed in individual pots. In order to constrain the extent of eyespan variation among males, only those with eyespan in the medium‐to‐large range between 6.95 and 7.94 mm were used (male eyespan typically vary between 4.3 and 8.6 mm under laboratory conditions). Inbred line females were discarded. Experimental female flies were drawn from the outbred population using the above methods. No constraint was placed on the range of eyespan of females used in the experiments.

### 2.3 Assays

The experiment was set up in blocks of 10 males and 10 females in a round‐robin design (Ingleby, Hunt, & Hosken, 2013; Mackay et al., 2005). All 10 males were tested with one of the 10 females within a bout (♂1 × ♀1, ♂2 × ♀2,…, ♂10 × ♀10) and then sequentially in the next bout with another female (♂1 × ♀2, ♂2 × ♀3,…, ♂10 × ♀1), until all males had been tested with all females (and vice versa). All observations in a block were made on a single day, with a total of 24 blocks, each having a unique set of males and females per block. By the nature of the round‐robin design, each male saw all females in a complementary preordained order, and thereby trial position effects of female or male traits, line, or inbreeding were avoided. The total sample size was 240 males (×10 females each) and 240 females (×10 males each) and hence 2,400 male–female pairs. The males were drawn from the 12 inbred lines and the outbred stock population. Only one male per line (or stock) was used per block, and by necessity not every line (or stock) was represented each day. Therefore, the total number of males per line over the whole experiment varied between 15 and 21. A round‐robin design was an efficient way to assess male and female behavior over several mating opportunities.

At the start of observations, a female was taken from her pot and introduced with a pooter into a male's pot. Males were given 15 min in which to make a mating attempt with a female. If an attempt was made, then the time was noted and acceptance (engagement of the male and female genitalia) or rejection (female abdominal shaking and kicking until the male is dislodged) recorded. Accepted matings were carefully interrupted using a pooter to dislodge the male before sperm transfer to maintain her virginity (<30 s; Cotton, Rogers, et al., 2006; Harley et al., 2013). If no attempt was made in 15 min, then the observation was concluded. Sometimes, the male made a poorly aimed mounting of the female that resulted in him falling off, in which case recording of male behavior continued until a successful mating attempt was made or 15 min had passed.

Three male behaviors were recorded, including whether a male followed the female (“follows”), moved his abdomen up and down (“bobs”), and engaged his legs with her legs or antennae (“grapples”). If a behavior continued for over ten seconds, then each further 10 s was recorded as a new event. We also recorded fights between males and females, but these were too infrequent for any further analysis (13 of 2,358 pairings). Each observer set ups 10 pairs to watch simultaneously. This made it impossible to accurately time exactly how long a male was performing a behavior, and hence, observers kept a tally every 10 s. The pots were kept next to each other and scanned in a line, allowing a large number to be assessed at the same time.

**Figure 1:**
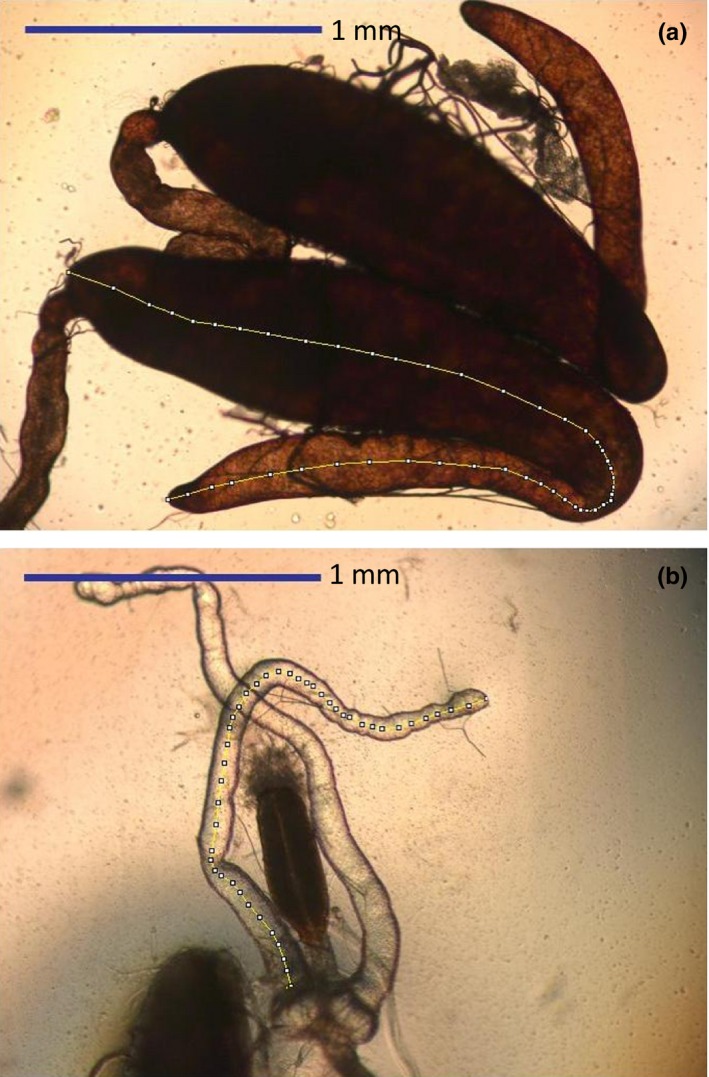
Testes (a) and accessory glands (b) of *Diasemopsis meigenii*. Testes and accessory glands are measured by tracing a midline (denoted by yellow line) that longitudinally bisects each organ

On completion of each block of observations, the males and females were frozen and their eyespan measured. Male eyespan had been constrained experimentally. Female eyespan varied and was taken as a measure of body size. In addition, male testes and accessory glands were dissected (Figure 1) and measured as the length of a line that bisected the middle of the testis or accessory gland to the nearest 0.01 mm (Rogers, Chapman, et al., 2005) in NIH ImageJ (Abramoff et al., 2004; Schneider et al., 2012; SI1). It was impossible to measure the accessory glands (41/240) and testes (15/240) of some individuals due to breakages during dissection, so such individuals were omitted from analyses of variation in male reproductive traits.

### 2.4 Statistical analysis

Statistics were performed in R (R Core Team 2013) using lme4 (version 3.2.5; Bates, Maechler, Bolker, & Walker, 2015), with some simple statistics derived from JMP version 11.2 (SAS Institute Inc., Cary, NC). Each male–female pair was coded as having shown a mating attempt (yes/no) and acceptance/rejection of the mating attempt. We ran tests of whether the number of mating attempts and acceptance given a mating attempt were related to female eyespan, and male eyespan, reproductive organ size, and behavior. These outcomes were modeled in generalized linear mixed effect models (GLMMs), with binomial error structure, fitted by maximum likelihood (Laplace approximation), with the logit link function. REML (or maximum likelihood) was used in linear mixed effect models of female (i.e., eyespan) and male traits (i.e., eyespan, reproductive organ size, and behavior). Models were compared using ANOVA which, along with random and fixed effect sizes, are reported in Supporting Information.

In all statistical tests, male identity (random effect) was nested within inbred line (random effect), as is standard for analyses of genetic lines (Lynch & Walsh, 1998). An analysis based on AICc supported the addition of female identity (random effect) and block (random effect) to improve model fit, and these two variables were added to all subsequent models. We initially tested whether mating attempts and acceptance (given a mating attempt) were affected by female eyespan. This was true for attempts but not for accepts (see Section 3) and so we included female eyespan as a covariate (fixed effect) in all further analyses of attempts.

Testes and accessory glands size were highly positively correlated (see Section 3). To reduce the dimensionality of the male reproductive measures, we used the first principal component of variation of these two traits (Pearson, 1901) calculated from the data set of inbred males (PC loading: testes 0.707, accessory glands 0.707, eigenvalue 1.409, 70.43% variance explained, χ^2^ = 38.64, *p* < .001, *N* = 183). A similar approach was taken for the behavioral measures. As the amount of behavior covaried with time taken to the mating attempt (when observation ceased), we calculated the principal components based on behavioral elements per second. PC1 reflected more behavior in general (PC1 loading: follows 0.678, bobs 0.476, grapples 0.560, eigenvalue 1.242, 41.41% variance explained, χ^2^ = 70.26, *p* < .001), and PC2 reflected antagonism between bobs and grapples (PC2 loading: follows −0.038, bobs 0.784, grapples −0.620, eigenvalue 0.964, 31.12% variance explained, χ^2^ = 13.05, *p* < .001; *N* = 1,495).

To test whether there was genetic variation among the 12 male inbred lines in the number of attempts and acceptance, we ran GLMMs similar in structure to those used above, including male identity (random effect) and female eyespan (fixed effect in mating attempts), as covariates. Additional covariates were added to these analyses where they had been shown to be important in previous analyses. We tested whether the addition of inbred line (random effect) improved model fit. In addition, trait values (male eyespan, reproductive organ size, and behavior) were subject to linear mixed effect models, testing for genetic variation between the inbred line males. These tests for genetic variation were then repeated including data from outbred males to determine whether inbreeding per se influenced the number of mating attempts and acceptance or trait values.

## 3 RESULTS

### 3.1 Female eyespan

**Figure 2:**
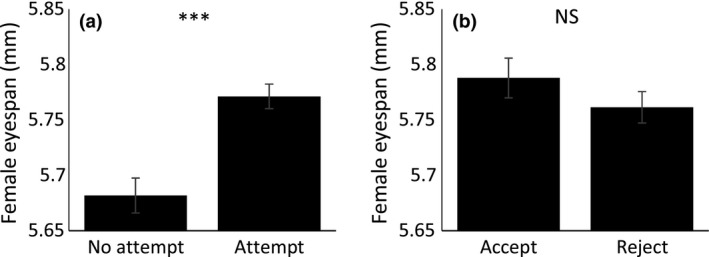
(a) Mean ± *SE* eyespan of females that did (Attempt) or did not (No attempt) receive a mating attempt and (b) accepted (Accept) or rejected (Reject) mating attempts; ***p < .001, **p < .01, *p < .05, NS: nonsignificant

There was considerable variation in the number of mating attempts received (range 2–10) and the number accepted by individual females (range 0–10). Female eyespan showed considerable variation (range 4.18–6.36 mm, mean ± *SD* 5.74 ± 0.444 mm, *N* = 238). Males attempted to mate more often with larger eyespan females (χ^2^ = 15.699, *p* < .001, *N* = 238 females). However, the mean eyespan of females that accepted male mating attempts did not differ from those who rejected mating attempts (χ^2^ = 15.699, *p* = .573, *N* = 238 females; Figure 2).

### 3.2 Male eyespan, reproductive organ size, and behavior

We controlled for male eyespan in the experimental design (range 6.95–7.94 mm, mean ± *SD* = 7.544 ± 0.258 mm, *N* = 224). The remaining variation in male eyespan was neither associated with the number of mating attempts (χ^2^ = 0.379, *p* = .538, *N* = 220 males) nor with the number of acceptances of mating attempts (χ^2^ = 1.263, *p* = .261, *N* = 213 males).

Nonetheless, there was variation in male reproductive organ size (testis range 2.664–4.232 mm, 3.405 ± 0.327 mm, *N* = 210; accessory glands range 1.001–1.878 mm, 1.409 ± 0.190 mm, *N* = 186). We combined these traits into a single principal component as the size of the two reproductive organs was strongly positively correlated (Pearson ρ_183_ = 0.401, *p* < .001, *N* = 183). Variation in male reproductive organ size was not associated with the number of mating attempts (χ^2^ = 0.166, *p* = .684, *N* = 183 males). There was a tendency for the number of acceptances of mating attempts to increase with reproductive organ size, but this relationship did not reach significance (χ^2^ = 2.972, *p* = .085, *N* = 177 males).

**Figure 3:**
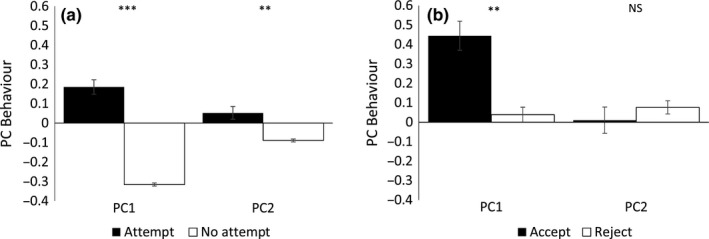
(a) Mean ± *SE* PC1 Behavior and PC2 Behavior of males that did (Attempt; Black) or did not (No attempt; White) make a mating attempt and (b) had their attempts accepted (Accept; Black) or rejected (Reject; White). ***p < .001, **p < .01, *p < .05, NS: nonsignificant

Male behavior directed at females was recorded in more than half of individual pairings (52.0%, *N* = 2,200). The behavioral traits occurred at different frequencies: follows in 688, bobs in 860, and grapples in 263 cases. The separate elements of behavior were combined into two principal components. In pairings that led to a mating attempt, males performed more PC1 behavior (χ^2^ = 190.440, *p* < .001, *N* = 2,186 matings) and more PC2 behavior (χ^2^ = 7.721, *p* = .005, *N* = 2,186 matings) compared to when they did not. A model with both PC1 and PC2 behaviors showed that both components of behavior were independently more intense when there was a mating attempt (χ^2^ = 44.563, *p* < .001, *N* = 2,186 matings). When the mating attempt was accepted, males performed more PC1 behavior (χ^2^ = 7.451, *p* = .006, *N* = 1,385 matings), but variation in PC2 behavior did not differ between accepted or rejected mating attempts (χ^2^ = 0.050, *p* = .823, *N* = 1,385 matings, Figure 3).

As male reproductive organ size was negatively associated with variation in PC1 behavior (χ^2^ = 7.451, *p* = .006, *N* = 1,385 matings), although not with PC2 behavior (χ^2^ = 0.050, *p* = .823, *N* = 1,385 matings), the analyses of behavior above were repeated with PC reproductive organ size as a control covariate. This did not change any of the observed relationships of PC1 or PC2 behavior with either mating attempts or acceptance of mating attempts (see Supporting Information).

### 3.3 Male genetic variation

**Figure 4:**
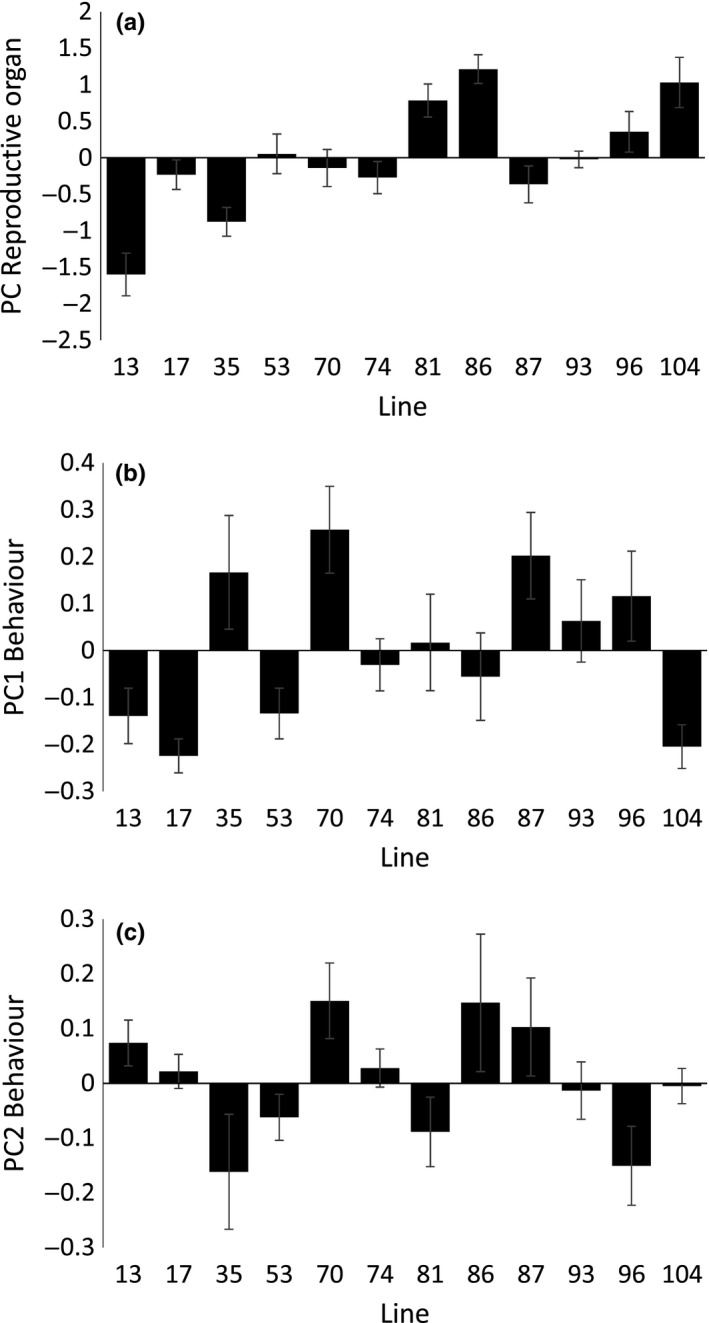
Mean ± *SE* (a) PC Reproductive organ, (b) PC1 Behavior and (c) PC2 Behavior for the 12 inbred lines

Inbred lines showed variation in male eyespan (χ^2^ = 9.883, *p* = .002, *N* = 12 lines), PC reproductive organ size (χ^2^ = 47.595, *p* < .001, *N* = 12 lines), PC1 behavior (χ^2^ = 5.866, *p* = .015, *N* = 12 lines), and PC2 behavior (χ^2^ = 4.295, *p* = .038, *N* = 12 lines; Figure 4). After adding covariates for the phenotypic traits that were important in previous analyses of mating attempts (PC1 and PC2 behavior) and acceptance (PC reproductive organ size and PC1 behavior), there was evidence of an effect of inbred line on mating attempts (χ^2^ = 7.451, *p* = .006, *N* = 12 lines) and on acceptance given a mating attempt (χ^2^ = 9.794, *p* = .002, *N* = 12 lines).

The focal inbred males were compared to stock outbred males. Inbred and outbred males did not differ in eyespan (χ^2^ = 0.756, *p* = .385, *N* = 236 males), PC reproductive organ size (χ^2^ = 0.408, *p* = .523, *N* = 196 males), PC1 behavior (χ^2^ = 0.004, *p* = .950, *N* = 240 males), or PC2 behavior (χ^2^ = 0.917, *p* = .338, *N* = 240 males). Outbred males did not make more mating attempts (χ^2^ = 0.065, *p* = .799, *N* = 240 males; mean ± *SE*. proportion attempts made within 900s, outbred = 0.67 ± 0.05, inbred = 0.63 ± 0.02) and nor were they accepted more frequently than inbred males (χ^2^ = 0.755, *p* = .385, *N* = 190 males; mean ± *SE* proportion accepts given an attempt, outbred = 0.32 ± 0.08, inbred = 0.36 ± 0.02).

## 4 DISCUSSION

Studies of sexual selection have emphasized the importance of mate preference for prominent, exaggerated sexual traits (Andersson, 1994; Davies, Krebs, & West, 2012; Maynard Smith & Harper, 2003). This is true of stalk‐eyed fly species, where research effort has focused on male eyespan, which is greatly expanded beyond that seen in females, and can even exceed body length (Baker & Wilkinson, 2003; Chapman et al., 2005). But as with other examples where sexual selection has produced highly exaggerated male characters, these are not the only traits that govern male–female interactions over mating (e.g., Aquiloni, Massolo, & Gherardi, 2009; Bro‐Jorgensen, 2010; Jones et al., 2014; Starnberger, Preininger, & Hodl, 2014). Here we investigated the importance of other male (testis and accessory gland sizes) and female (eyespan) morphological traits, as well as male behaviors that precede mating and might be elements of male courtship. These additional traits were assessed through two outcome variables, namely male mating attempts and female acceptance or rejection of such mating attempts. In addition, we looked for evidence of genetic variation underlying the male characters as well as affecting mating decisions, using a suite of inbred lines, in comparison with outbred control flies.

In order to study these additional traits, we adopted a round‐robin design in which 240 males were paired with 10 females each, and in parallel 240 females were paired with 10 males. The advantage of this approach was that it was feasible to analyze a very large number of male–female pairings, 2,400 in total. Each observer monitored up to 10 pairs at a time, for a maximum of 15 min each, and so could track the 10 × 10 sets in each round‐robin block within a reasonable experimental period per day (150‐minute observation plus setup and handling time). This meant that the intensity of study of males and females was equal—an advantage given that we were studying male mating attempts and female acceptance or rejection of those attempts. The large‐scale approach was in particular useful in the behavioral analysis, as behavioral elements were not observed in all interactions and sometimes infrequently. The design did force a compromise, as behaviors were measured in 10‐second intervals rather than as continuous variables. This potentially could be overcome by making video recordings for subsequent analysis. We recommend the round‐robin design for future studies of mating behavior.

We found that males preferred attempting to mate with large eyespan females. Such male choice echoes the finding that males mate for longer and ejaculate more sperm when mating with large eyespan females (Harley et al., 2013). In addition, in the related stalk‐eyed fly species *T. dalmanni*, males show preference for large eyespan females, both in the wild and under controlled laboratory conditions (Cotton et al., 2015). The likely reason for male preference in both these stalk‐eyed fly species is that female eyespan strongly correlates positively with fecundity (Cotton et al., 2015; Harley et al., 2013). Similar male preference for female traits that are good predictors of fecundity has been observed in a range of species, typically for traits correlated with female size (Bonduriansky, 2001) and female ornament size, when ornaments are female specific (Amundsen 2000, Amundsen and Forsgren, 2001) or exaggerated in both sexes (Baldauf, Bakker, Kullmann, & Thünken, 2011; Doutrelant et al., 2008; Potti, Canal, & Serrano, 2013). This finding does not identify the character used by males, as female eyespan is tightly correlated with body size and other morphological traits both in *D. meigenii* and other stalk‐eyed fly species (Cotton et al., 2004b, 2015; Harley et al., 2013).

We expected that males with larger reproductive organs would make more mating attempts because they are less constrained in their reproductive resources (Dewsbury, 1982; Moore, Harris, Montrose, Levin, & Moore, 2004; Preston, Stevenson, Pemberton, & Wilson, 2001; Rogers, Chapman, et al., 2005). However, there was no relationship between the number of mating attempts and reproductive organ size (the first principal component of accessory gland size and testis size). Our results suggest that male propensity to mate is independent of variation in reproductive organ size. A simple explanation is that copulations were interrupted before sperm transfer in our experiments. As a result, males did not suffer ejaculate depletion and their accessory glands and testes remained fully laden throughout the experiment. Presumably, if we had allowed ejaculation to take place, males with larger reproductive organs would have been able to make more mating attempts. This notion is supported by the observation in *T. dalmanni* that accessory gland size (but not testis size) is phenotypically and genetically related to mating frequency (Baker et al., 2003; Rogers, Baker, et al., 2005; Rogers, Chapman, et al., 2005).

As females control acceptance, we did not expect that females would be more likely to accept mating attempts from males with larger reproductive organs. Females can knock males off through violent body shaking and avoid engagement with male genitalia by ovipositor extension (Cotton, Rogers, et al., 2006). Yet there was a strong tendency toward males with larger reproductive organs being accepted more frequently once a mating attempt had been initiated. A similar result has been reported in *Drosophila melanogaster*, where males with large accessory glands were accepted for copulation more frequently (Bangham, Chapman, & Partridge, 2002). It seems unlikely that females directly identified males with large testes and accessory glands via visual assessment. While the reproductive organs of adult stalk‐eyed flies grow and vary in size with age and nutritional status (in *T. dalmanni*; Baker et al., 2003; Rogers et al., 2008), adult fly external body size and shape are fixed at eclosion (Buschbeck, Roosevelt, & Hoy, 2001). Indirect evaluation mechanisms are plausible. Under normal conditions, females could have used the male ornament and body size, which are positively correlated with internal reproductive organ size (Cotton et al., 2010; Rogers et al., 2008), as is the case in several other insect species (Bangham et al., 2002; Fairn, Schulte‐Hostedde, & Yves, 2007; Oh, Kim, Yoon, Kim, & Kim, 2014). But in our experimental design any information from male eyespan was explicitly constrained so that it could not serve as a proxy for male reproductive organ size (these traits were not correlated, Pearson ρ_183_ = −0.031, *p* = .682). Similarly for *D. melanogaster,* the greater success of males with large accessory glands was evident after controlling for body size (Bangham et al., 2002). Premating male display is a possible signal (see below), but there was no consistent association of male behavior with reproductive organ size (see Supporting Information). Other possibilities are male contact pheromones (e.g., Starnberger et al., 2014) or female detection of differences in weight once the male has mounted (Schlaepger & McNeil, 2000). These hypotheses have not been investigated in stalk‐eyed flies. Our results suggest that there are additional cues used by females to assess males that attempt to mate with them, and they are worthy of further investigation.

In this study, we tracked a diversity of male behaviors that could be construed as “courtship.” The behaviors involved the male following behind a female, bobbing his abdomen and engaging his legs and antennae with hers. These behaviors are not exaggerated but are a definite and repeated feature of male activity in the presence of females. But we note that in about half of male–female pairs, a mating attempt occurred without any behavior at all and in many cases led to acceptance by the female. Although the mean time to mating in *D. meigenii* was several minutes (mean ± *SD* = 288.46 ± 247.31 s), male–female interactions prior to mating can be very brief (lower bound of range 10 s). So these behaviors cannot be construed as necessary courtship signals that inevitably precede a mating attempt or acceptance by the female. Nonetheless, males that performed more behaviors per unit time made more mating attempts. This was true for both principal components, the first which simply reflected more behavior (PC1) and the second which reflected an antagonism between more abdomen bobbing and less “talking” engagement of antennae and legs (PC2). In addition, males that performed more behaviors (PC1 only) were more likely to be accepted after mounting; this difference suggests that all of the behavioral components are signals that females take into account, and the greater weight on abdomen bobbing versus “grapples” was of lesser importance. However, the information content of these behaviors is not obvious. It is possible that they contribute to mutual coordination between male and female. But this is hard to assess because unlike many other species (Faggioni et al., 2017; Marshall‐Ball, Mann, & Slater, 2006; Soma & Iwama, 2017) there was no evident reciprocal female behavior toward the male. Although one could envisage that the male behaviors may incur some energetic costs, it is unlikely that they act as “handicaps,” creating a condition‐dependent cost for the male that performs them (Iwasa, Pomiankowski, & Nee, 1991; Kuijper, Pen, & Weissing, 2012). So it seems unlikely they are signals of male quality.

We also compared the performance of males from a set of inbred lines to assess genetic variation underlying mating outcomes. There was genetic (between‐line) variation in all of the male traits measured: eyespan, reproductive organ size, and behavior (both principal components). There was also genetic variation underlying the rate at which males made mating attempts and in the rate of female acceptance of male mating attempts. The latter tests were carried out with appropriate control for those phenotypic traits already shown to have importance in mating attempts (PC1 and PC2 behavior) and acceptance (PC reproductive organ size and PC1 behavior), indicating that there was genetic variation in these outcomes in addition to that relating to the morphological and behavioral trait genetic differences between lines. This indicates that other unmeasured features contributed to the outcome of male–female mating interactions. These results point to additional female benefits, as paternal genetic variation in testis and accessory gland size, as well as in behavior elicited during mating interactions, will be inherited and so potentially contribute to the reproductive success of sons in the next generation. Whether these genetic consequences are connected to “good genes” viability benefits to both male and female offspring (Iwasa et al., 1991; Kuijper et al., 2012) cannot be resolved from this work. It should be noted that the genetic lines used in the experiments represent an initial random sample of genetic variation from our stock population of *D. meigenii*, filtered through several generations of brother–sister inbreeding, which will have eliminated deleterious alleles (Bellamy, Fowler, & Pomiankowski, 2014; Bellamy et al., 2013). Of the >100 lines initiated, around 20% survived the initial period of intense inbreeding, in part because of the uncovering of recessive deleterious alleles (Bellamy et al., 2013). This culling and subsequent adaptive change may explain the lack of evidence of inbreeding depression in traits, attempts, or acceptance when the inbred lines were compared to outbred control males. In natural populations, there is likely to be much more segregating genetic variation with larger consequences for sexual selection than demonstrated here for a suite of laboratory‐adapted inbred lines.

In addition, we hypothesized that differences in eyespan among females might affect their propensity to mate. Female eyespan is positively correlated with female size and fecundity, and more fecund females may need to mate more often to secure their fertility (Cotton, Rogers, et al., 2006; Harley et al., 2013). However, female eyespan did not alter female acceptance or rejection of male mating attempts, a pattern that contrasted with male mating attempts which were more commonly directed at females with larger eyespan (see above). The former outcome is consistent with previous mate choice studies using *D. meigenii* that also found no association of female eyespan with the rejection rate (Cotton, Rogers, et al., 2006; Harley et al., 2013). In all of these studies, the experimental design kept females unmated with the benefit that all females had similar mating experience. However, had females been allowed to mate, then differences related to female eyespan might have been revealed. Females need to mate repeatedly in order to maintain fertility (Baker et al., 2001; Harley, Fowler, & Cotton, 2010; Rogers, Grant, Chapman, Pomiankowski, & Fowler, 2006), so it is likely that larger females need to mate more often to gain sufficient sperm. On the other hand, large females have higher reproductive value and attract greater male investment per ejaculate and so may need to mate less often than smaller females (Harley et al., 2013). These patterns associated with female eyespan merit further investigation.

In conclusion, animals evolve sophisticated means of assessing the quality of potential mates and signaling to members of the opposite sex in order to gain mating opportunities. Many sexual traits and displays are exaggerated and elaborated and have rightly attracted attention. But in order to form a more comprehensive understanding of sexual selection, it is important to determine the degree to which other factors play a role in mate choice. We have shown in *D. meigenii* that a range of behavioral cues is important in mate choice, even though they are not a necessary component of male courtship. In addition, females are sensitive to male reproductive organ size (testes and accessory glands) when choosing among males. Furthermore, our study of inbred lines showed that genetic differences underlie variation between males in their readiness to mate and their attractiveness to females. These additional traits, in concert with male eyespan (and genetic variation in that trait), contribute to the benefits of mate choice in the stalk‐eyed fly.

## CONFLICT OF INTEREST

None declared.

## AUTHOR CONTRIBUTIONS

NCC, KF, and AP conceived and designed the experiment. KF, LB, NCC, AT, and JH contributed to the breeding of inbred lines. NCC and JH produced flies for the experiment. NCC measured male and female eyespan. NCC and PS carried out the experiment. PS performed dissections and measured male testes and accessory gland length. AP and NCC performed the statistics. NCC, KF, and AP wrote the manuscript with input from JH and LB.

## DATA ACCESSIBILITY

The data has been archived in Dryad https://doi.org/10.5061/dryad.7dd43. R scripts are available as online supporting information.
