# Supplementary material for: The complexity of mating decisions in stalk‐eyed flies
Source: Ecol Evol. 2017 Jul 18;7(17):6659–68. doi: 10.1002/ece3.3225 (PMC5587473; doi:10.1002/ece3.3225)
Supplement: Supplementary file 1 [file ECE3-7-6659-s001.docx]

**Supplementary Information**

Each male-female pair was coded as having shown a mating attempt (yes/no) and acceptance/rejection of the mating attempt. We ran tests of whether the number of mating attempts and acceptance given a mating attempt were related to female eyespan, and male eyespan, reproductive organ size and behaviour. These outcomes were modelled in generalised linear mixed effects models (GLMM), with binomial error structure, fitted by maximum likelihood (Laplace Approximation), with the logit link function. REML (or Maximum Likelihood) was used in linear mixed effects models of female (i.e. eyespan) and male traits (i.e. eyespan, reproductive organ size, behaviour).

Models were compared using ANOVA for which P-values are reported. The two models compared are arbitrarily labelled model0 and model1, with the latter being the model with a greater number of effects. In addition, we report random and fixed effect sizes for model1. Statistics were computed in R version 3.2.4 (R Development Core Team, 2016), using the lme4 package (Bates, Mächler, Bolker, & Walker, 2015). Sometimes models of behaviour generated warnings of failure to converge. In this case, we used a different optimizer BOBYQA (bound optimization by quadratic approximation; Powell 2009), and indicate this in the text.

Significance codes: ‘***’ 0.001 ‘**’ 0.01 ‘*’ 0.05 ‘.’ 0.1.

R code designations:

(1|FID) – FID is a random effect

(1|LINE/MID) – MID is nested within LINE, and both are random effects

| Abbreviations: |  |
| --- | --- |
| LINE | genetic line |
| in/out | inbred or outbred |
| MID | male ID |
| FID | female ID |
| BLOCK | round robin block structure of the data |
| Female.ES | female eyespan |
| Male.ES | male eyespan |
| PC.Reporgan | principal component of variation in testes and accessory gland size |
| PC1Beh.min | first principal component of variation of behavioural components follows, bobs and grapples per minute |
| PC2Beh.min | second principal component of variation of behavioural components follows, bobs and grapples per minute |

**Supplementary References**

Bates, D., Mächler, M., Bolker, B., & Walker, S. (2015). Fitting linear mixed-effects models using lme4. *Journal of Statistical Software*, 67, 1.

Powell, M. J. D. (2009). [The BOBYQA algorithm for bound constrained optimization without derivatives](http://www.damtp.cam.ac.uk/user/na/NA_papers/NA2009_06.pdf). Department of Applied Mathematics and Theoretical Physics, Cambridge University. <http://www.damtp.cam.ac.uk/user/na/NA_papers/NA2009_06.pdf>

R Development Core Team. (2016). R: A Language and Environment for Statistical Computing. *R Foundation for Statistical Computing Vienna Austria*. <http://www.R-project.org/>

**Index**

**BASIC MODEL page 4**

**a) Attempts**

**b) Accepts**

**FEMALE EYESPAN page 5**

**a) Attempts**

**b) Accepts**

**MALE EYESPAN, REPRODUCTIVE ORGAN SIZE AND BEHAVIOUR**

**1. Male eyespan page 6**

**a) Attempts**

**b) Accepts**

**2. Male reproductive organ size page 7**

**a) Attempts**

**b) Accepts**

**3. Male Behaviour page 8**

**a) Attempts**

**b) Accepts**

**REPRODUCTIVE ORGAN SIZE AS A CONTROL COVARIATE**

**1. Behaviour dependent on reproductive organ size page 12**

**PC1Beh.min, PC2Beh.min**

**2. Male Behaviour page 13**

**a) Attempts**

**b) Accepts**

**GENETIC VARIATION IN MALES (AMONG INBRED LINES)**

**1. Trait differences page 15**

**Male.ES, Reproductive Organ Size, Behaviour**

**2. Outcomes page 16**

**a) Attempts**

**b) Accepts**

**GENETIC VARIATION IN MALES (INBREEDING STATUS)**

**1. Trait differences page 18**

**Male.ES, Reproductive Organ Size, Behaviour**

**2. Outcomes page 19**

**a) Attempts**

**b) Accepts**

**BASIC MODEL**

In a preliminary analysis we tested the sequential addition of a variety of random factors on mating attempts and on acceptance given a mating attempt. All factors improved model fit (except BLOCK in attempts) and were included in further models. Here all models are listed (i.e. model0….model3) along with sequential comparisons by ANOVA (i.e. model0 vs. model1, model1 vs.model2 etc.). Random effect sizes are given for model3.

**a) Attempts**

model0: AT ~ (1 | LINE)

model1: AT ~ (1 | LINE/MID)

model2: AT ~ (1 | LINE/MID) + (1 | FID)

model3: AT ~ (1 | LINE/MID) + (1 | FID) + (1 | BLOCK)

Df AIC Chisq Pr(>Chisq)

model0 2 2879.6

model1 3 2722.8 158.8491 < 2.2e-16 ***

model2 4 2710.3 14.4406 0.0001447 ***

model3 5 2709.7 2.5967 0.1070880

Random effects:

Groups Name Variance Std.Dev.

FID (Intercept) 0.24333 0.4933

MID:LINE (Intercept) 1.03121 1.0155

BLOCK (Intercept) 0.11960 0.3458

LINE (Intercept) 0.07263 0.2695

Number of obs: 2200, groups: FID, 240; MID:LINE, 224; BLOCK, 24; LINE, 12

**b) Accepts**

Models:

model0: AR ~ (1 | LINE)

model1: AR ~ (1 | LINE/MID)

model2: AR ~ (1 | LINE/MID) + (1 | FID)

model3: AR ~ (1 | LINE/MID) + (1 | FID) + (1 | BLOCK)

Df AIC Chisq Pr(>Chisq)

model0 2 1754.8

model1 3 1666.0 90.791 < 2.2e-16 ***

model2 4 1575.2 92.731 < 2.2e-16 ***

model3 5 1552.7 24.580 7.13e-07 ***

Random effects:

Groups Name Variance Std.Dev.

FID (Intercept) 1.4706 1.213

MID:LINE (Intercept) 1.0334 1.017

BLOCK (Intercept) 1.0529 1.026

LINE (Intercept) 0.7057 0.840

Number of obs: 1385, groups: FID, 240; MID:LINE, 217; BLOCK, 24; LINE, 12

**FEMALE EYESPAN**

As Female.ES had a significant effect on Attempts (but not on Accepts), it was included in all further models of Attempts (but not of Accepts)

**a) Attempts**

model0: AT ~ (1 | LINE/MID) + (1 | FID) + (1 | BLOCK)

model1: AT ~ Female.ES + (1 | LINE/MID) + (1 | FID) + (1 | BLOCK)

Chisq = 15.699, P = 7.427e-05 ***

Random effects:

Groups Name Variance Std.Dev.

FID (Intercept) 0.19313 0.4395

MID:LINE (Intercept) 1.04146 1.0205

BLOCK (Intercept) 0.10476 0.3237

LINE (Intercept) 0.07474 0.2734

Number of obs: 2186, groups: FID, 238; MID:LINE, 224; BLOCK, 24; LINE, 12

Fixed effects:

Estimate Std. Error z value Pr(>|z|)

(Intercept) -2.9846 0.9187 -3.249 0.00116 **

Female.ES 0.6387 0.1586 4.028 5.64e-05 ***

(note: positive value means trait higher when there is an Attempt)

**b) Accepts**

model0: AR ~ (1 | LINE/MID) + (1 | FID) + (1 | BLOCK)

model1: AR ~ Female.ES + (1 | LINE/MID) + (1 | FID) + (1 | BLOCK)

Chisq = 0.3178, P = 0.5729

Random effects:

Groups Name Variance Std.Dev.

FID (Intercept) 1.4997 1.2246

MID:LINE (Intercept) 1.0920 1.0450

BLOCK (Intercept) 1.0352 1.0174

LINE (Intercept) 0.6993 0.8362

Number of obs: 1379, groups: FID, 238; MID:LINE, 217; BLOCK, 24; LINE, 12

Fixed effects:

Estimate Std. Error z value Pr(>|z|)

(Intercept) 2.1020 1.8301 1.149 0.251

Female.ES -0.1787 0.3119 -0.573 0.567

(note: negative value means trait higher when the mating attempt is Accepted)

**MALE EYESPAN, REPRODUCTIVE ORGAN SIZE AND BEHAVIOUR**

**1. Male eyespan**

**a) Attempts**

model0: AT ~ Female.ES + (1 | LINE/MID) + (1 | FID) + (1 | BLOCK)

model1: AT ~ Male.ES + Female.ES + (1 | LINE/MID) + (1 | FID) + (1 | BLOCK)

Chisq = 0.3788 P = 0.5383

Random effects:

Groups Name Variance Std.Dev.

FID (Intercept) 0.21986 0.4689

MID:LINE (Intercept) 1.01139 1.0057

BLOCK (Intercept) 0.11627 0.3410

LINE (Intercept) 0.07733 0.2781

Number of obs: 2146, groups: FID, 238; MID:LINE, 220; BLOCK, 24; LINE, 12

Fixed effects:

Estimate Std. Error z value Pr(>|z|)

(Intercept) -4.5432 2.8556 -1.591 0.112

Male.ES 0.2120 0.3534 0.600 0.549

Female.ES 0.6321 0.1620 3.902 9.55e-05 ***

(note: positive value means trait higher when there is an Attempt)

**b) Accepts (no Female.ES covariate)**

model0: AR ~ (1 | LINE/MID) + (1 | FID) + (1 | BLOCK)

model1: AR ~ Male.ES + (1 | LINE/MID) + (1 | FID) + (1 | BLOCK)

Chisq = 1.2625 P = 0.2612

Random effects:

Groups Name Variance Std.Dev.

FID (Intercept) 1.4751 1.2145

MID:LINE (Intercept) 1.0490 1.0242

BLOCK (Intercept) 1.0629 1.0310

LINE (Intercept) 0.6641 0.8149

Number of obs: 1362, groups: FID, 240; MID:LINE, 213; BLOCK, 24; LINE, 12

Fixed effects:

Estimate Std. Error z value Pr(>|z|)

(Intercept) -2.8414 3.4137 -0.832 0.405

Male.ES 0.5178 0.4501 1.150 0.250

(note: negative value means trait higher when the mating attempt is Accepted)

**2. Male reproductive organ size**

**a) Attempts**

model0: AT ~ Female.ES + (1 | LINE/MID) + (1 | FID) + (1 | BLOCK)

model1: AT ~ PC.Reporgan + Female.ES + (1 | LINE/MID) + (1 | FID) + (1 | BLOCK)

Chisq = 0.1658 P = 0.6838

Random effects:

Groups Name Variance Std.Dev.

FID (Intercept) 0.16542 0.4067

MID:LINE (Intercept) 1.07096 1.0349

BLOCK (Intercept) 0.08079 0.2842

LINE (Intercept) 0.12126 0.3482

Number of obs: 1791, groups: FID, 238; MID:LINE, 183; BLOCK, 24; LINE, 12

Fixed effects:

Estimate Std. Error z value Pr(>|z|)

(Intercept) -2.92895 0.97572 -3.002 0.002684 **

PC.Reporgan -0.03956 0.09586 -0.413 0.679831

Female.ES 0.62684 0.16829 3.725 0.000195 ***

(note: positive value means trait higher when there is an Attempt)

**b) Accepts (no Female.ES covariate)**

model0: AR ~ (1 | LINE/MID) + (1 | FID) + (1 | BLOCK)

model1: AR ~ PC.Reporgan + (1 | LINE/MID) + (1 | FID) + (1 | BLOCK)

Chisq = 2.9719 P = 0.08472

Random effects:

Groups Name Variance Std.Dev.

FID (Intercept) 1.3512 1.1624

MID:LINE (Intercept) 0.9810 0.9905

BLOCK (Intercept) 1.2749 1.1291

LINE (Intercept) 0.4016 0.6337

Number of obs: 1136, groups: FID, 238; MID:LINE, 177; BLOCK, 24; LINE, 12

Fixed effects:

Estimate Std. Error z value Pr(>|z|)

(Intercept) 1.0873 0.3306 3.289 0.0010 **

PC.Reporgan -0.2390 0.1361 -1.756 0.0791 .

(note: negative value means trait higher when the mating attempt is Accepted)

**3. Male Behaviour**

**a) Attempts**

**PC1Beh.min (BOBYQA)**

model0: AT ~ Female.ES + (1 | LINE/MID) + (1 | FID) + (1 | BLOCK)

model1: AT ~ PC1Beh.min + Female.ES + (1 | LINE/MID) + (1 | FID) +

(1 | BLOCK)

Chisq = 190.44 P < 2.2e-16 ***

Random effects:

Groups Name Variance Std.Dev.

FID (Intercept) 0.1962 0.4429

MID:LINE (Intercept) 0.9994 0.9997

BLOCK (Intercept) 0.1150 0.3391

LINE (Intercept) 0.1364 0.3694

Number of obs: 2186, groups: FID, 238; MID:LINE, 224; BLOCK, 24; LINE, 12

Fixed effects:

Estimate Std. Error z value Pr(>|z|)

(Intercept) -1.9352 0.9602 -2.016 0.04385 *

PC1Beh.min 1.7869 0.1833 9.751 < 2e-16 ***

Female.ES 0.5131 0.1649 3.111 0.00186 **

(note: positive value means trait higher when there is an Attempt)

**PC2Beh.min (BOBYQA)**

model0: AT ~ Female.ES + (1 | LINE/MID) + (1 | FID) + (1 | BLOCK)

model1: AT ~ PC2Beh.min + Female.ES + (1 | LINE/MID) + (1 | FID) +

(1 | BLOCK)

Chisq = 7.7212 P = 0.005458 **

Random effects:

Groups Name Variance Std.Dev.

FID (Intercept) 0.19794 0.4449

MID:LINE (Intercept) 1.01757 1.0087

BLOCK (Intercept) 0.10842 0.3293

LINE (Intercept) 0.07458 0.2731

Number of obs: 2186, groups: FID, 238; MID:LINE, 224; BLOCK, 24; LINE, 12

Fixed effects:

Estimate Std. Error z value Pr(>|z|)

(Intercept) -2.98100 0.92217 -3.233 0.00123 **

PC2Beh.min 0.17964 0.06772 2.653 0.00798 **

Female.ES 0.63886 0.15922 4.013 6.01e-05 ***

(note: positive value means trait higher when there is an Attempt)

**PC1Beh.min + PC2Beh.min (BOBYQA)**

**Combination of both traits, examine by AICc**

model0 AT ~ Female.ES+ (1 | LINE/MID) + (1 | FID) + (1 | BLOCK)

model1 AT ~ Female.ES+ PC1Beh.min + (1 | LINE/MID) + (1 | FID) + (1 | BLOCK)

model2 AT ~ Female.ES+ PC2Beh.min + (1 | LINE/MID) + (1 | FID) + (1 | BLOCK)

model3 AT ~ Female.ES+ PC1Beh.min + PC2Beh.min + (1 | LINE/MID) + (1 | FID) + (1 | BLOCK)

df AICc

model0 6 2673.156

model1 7 2484.729

model2 7 2667.448

model3 8 2442.181

anova(model1,model3)

Chisq = 44.563, P = 2.463e-11 ***

Report model3

Random effects:

Groups Name Variance Std.Dev.

FID (Intercept) 0.2224 0.4716

MID:LINE (Intercept) 0.9222 0.9603

BLOCK (Intercept) 0.1088 0.3299

LINE (Intercept) 0.1545 0.3931

Number of obs: 2186, groups: FID, 238; MID:LINE, 224; BLOCK, 24; LINE, 12

Fixed effects:

Estimate Std. Error z value Pr(>|z|)

(Intercept) -1.8507 0.9870 -1.875 0.06078 .

Female.ES 0.5231 0.1695 3.085 0.00203 **

PC1Beh.min 2.0431 0.1996 10.235 < 2e-16 ***

PC2Beh.min 1.3272 0.2000 6.634 3.26e-11 ***

Summary: Combination of PC1Beh.min and PC2Beh.min produces a better fit.

**b) Accepts (no Female.ES covariate)**

**PC1Beh.min**

model0: AR ~ (1 | LINE/MID) + (1 | FID) + (1 | BLOCK)

model1: AR ~ PC1Beh.min + (1 | LINE/MID) + (1 | FID) + (1 | BLOCK)

Chisq = 7.4508 P = 0.006341 **

Random effects:

Groups Name Variance Std.Dev.

FID (Intercept) 1.3895 1.1788

MID:LINE (Intercept) 1.0210 1.0104

BLOCK (Intercept) 0.9511 0.9753

LINE (Intercept) 0.7282 0.8533

Number of obs: 1385, groups: FID, 240; MID:LINE, 217; BLOCK, 24; LINE, 12

Fixed effects:

Estimate Std. Error z value Pr(>|z|)

(Intercept) 1.09292 0.34917 3.130 0.00175 **

PC1Beh.min -0.16520 0.06323 -2.613 0.00899 **

(note: negative value means trait higher when the mating attempt is Accepted)

**PC2Beh.min**

model0: AR ~ (1 | LINE/MID) + (1 | FID) + (1 | BLOCK)

model1: AR ~ PC2Beh.min + (1 | LINE/MID) + (1 | FID) + (1 | BLOCK)

Chisq = 0.05 P = 0.8231

Random effects:

Groups Name Variance Std.Dev.

FID (Intercept) 1.4701 1.212

MID:LINE (Intercept) 1.0353 1.017

BLOCK (Intercept) 1.0496 1.025

LINE (Intercept) 0.7056 0.840

Number of obs: 1385, groups: FID, 240; MID:LINE, 217; BLOCK, 24; LINE, 12

Fixed effects:

Estimate Std. Error z value Pr(>|z|)

(Intercept) 1.07212 0.34903 3.072 0.00213 **

PC2Beh.min 0.01451 0.06286 0.231 0.81740

(note: negative value means trait higher when the mating attempt is Accepted)

**PC1Beh.min + PC2Beh.min**

**Combination of both traits, examine by AICc**

model0: AR ~ (1 | LINE/MID) + (1 | FID) + (1 | BLOCK)

model1: AR ~ PC1Beh.min + (1 | LINE/MID) + (1 | FID) + (1 | BLOCK)

model2: AR ~ PC2Beh.min + (1 | LINE/MID) + (1 | FID) + (1 | BLOCK)

model3: AR ~ PC1Beh.min + PC2Beh.min + (1 | LINE/MID) + (1 | FID) + (1 | BLOCK)

df AICc

model0 5 1552.698

model1 6 1547.264

model2 6 1554.665

model3 7 1548.273

anova(model1,model3)

Chisq = 0.0637 P = 0.8007

Random effects:

Groups Name Variance Std.Dev.

FID (Intercept) 1.3919 1.1798

MID:LINE (Intercept) 1.0201 1.0100

BLOCK (Intercept) 0.9548 0.9772

LINE (Intercept) 0.7301 0.8544

Number of obs: 1385, groups: FID, 240; MID:LINE, 217; BLOCK, 24; LINE, 12

Fixed effects:

Estimate Std. Error z value Pr(>|z|)

(Intercept) 1.09457 0.34969 3.130 0.00175 **

PC1Beh.min -0.16758 0.06350 -2.639 0.00831 **

PC2Beh.min -0.01866 0.07378 -0.253 0.80034

Summary: Adding both traits does not improve model fit (this is not surprising as PC2Beh.min alone is NS)

**REPRODUCTIVE ORGAN SIZE AS A CONTROL COVARIATE**

In these linear mixed models, we consider whether male behaviour is in part explained by male reproductive organ size. They were fitted using maximum likelihood. A negative effect size indicates that larger organ size is associated with less behaviour.

**1. Behaviour dependent on reproductive organ size**

**PC1Beh.min**

model0: PC1Beh.min ~ Female.ES + (1 | LINE/MID) + (1 | FID) + (1 |

BLOCK)

model1: PC1Beh.min ~ PC.Reporgan + Female.ES + (1 | LINE/MID) + (1 |

FID) + (1 | BLOCK)

Chisq = 3.9753 P = 0.04617 *

Random effects:

Groups Name Variance Std.Dev.

FID (Intercept) 0.06025 0.2455

MID:LINE (Intercept) 0.02042 0.1429

BLOCK (Intercept) 0.05022 0.2241

LINE (Intercept) 0.01372 0.1171

Residual 1.10557 1.0515

Number of obs: 1791, groups: FID, 238; MID:LINE, 183; BLOCK, 24; LINE, 12

Fixed effects:

Estimate Std. Error df t value Pr(>|t|)

(Intercept) -0.83624 0.46908 235.28000 -1.783 0.0759 .

Female.ES 0.14199 0.08091 238.66000 1.755 0.0806 .

PC.Reporgan -0.05503 0.02765 124.24000 -1.990 0.0488 *

**PC2Beh.min**

model0: PC2Beh.min ~ Female.ES + (1 | LINE/MID) + (1 | FID) + (1 |

BLOCK)

model1: PC2Beh.min ~ PC.Reporgan + Female.ES + (1 | LINE/MID) + (1 |

FID) + (1 | BLOCK)

Chisq = 5032.8 5e-04 P = 0.9821

Random effects:

Groups Name Variance Std.Dev.

FID (Intercept) 0.032414 0.18004

MID:LINE (Intercept) 0.000000 0.00000

BLOCK (Intercept) 0.000000 0.00000

LINE (Intercept) 0.006313 0.07945

Residual 0.940687 0.96989

Number of obs: 1791, groups: FID, 238; MID:LINE, 183; BLOCK, 24; LINE, 12

Fixed effects:

Estimate Std. Error df t value Pr(>|t|)

(Intercept) 6.936e-02 3.388e-01 2.598e+02 0.205 0.838

Female.ES -1.033e-02 5.870e-02 2.585e+02 -0.176 0.860

PC.Reporgan -3.519e-04 2.212e-02 2.016e+02 -0.016 0.987

**2. Male Behaviour**

Here we added PC reproductive organ size as a control covariate to the analysis of male behaviour as an explanatory effect of mating attempts and acceptance. Rather than repeating all analyses, we report AICc values for each model combination with PC1 and PC2 behaviour per minute, and then show an ANOVA comparing the two lowest AICc values, along with random/fixed effect sizes for the best model. The main point here is to show that the inclusion of PC reproductive organ size as a control covariate does not alter any of the previous conclusions about male behaviour and attempts or accepts.

**a) Attempts (BOBYQA)**

**(reproductive organ size added as a covariate)**

model0: AT ~ PC.Reporgan + Female.ES

model1: AT ~ PC.Reporgan + Female.ES + PC1Beh.min

model2: AT ~ PC.Reporgan + Female.ES + PC2Beh.min

model3: AT ~ PC.Reporgan + Female.ES + PC1Beh.min + PC2Beh.min

df AICc

model0 7 2189.273

model1 8 2031.541

model2 8 2181.897

model3 9 1992.313

anova(model1,model3)

Chisq = 41.249, P = 1.34e-10 ***

Report model3

Random effects:

Groups Name Variance Std.Dev.

FID (Intercept) 0.2341 0.4839

MID:LINE (Intercept) 1.0030 1.0015

BLOCK (Intercept) 0.1038 0.3222

LINE (Intercept) 0.1812 0.4257

Number of obs: 1791, groups: FID, 238; MID:LINE, 183; BLOCK, 24; LINE, 12

Fixed effects:

Estimate Std. Error z value Pr(>|z|)

(Intercept) -1.768640 1.080291 -1.637 0.10159

PC.Reporgan 0.001185 0.099719 0.012 0.99052

Female.ES 0.515250 0.185457 2.778 0.00546 **

PC1Beh.min 2.134513 0.228358 9.347 < 2e-16 ***

PC2Beh.min 1.438051 0.229217 6.274 3.52e-10 ***

Summary: With model3, there is no difference to a model without PC.Reporgan included as a covariate, as both PC1Beh.min and PC2Beh.min remain important.

**b) Accepts (BOBYQA)**

**(reproductive organ size added as a covariate; no Female.ES covariate)**

Combination of traits, examine by AICc

model0: AR ~ PC.Reporgan

model1: AR ~ PC.Reporgan + PC1Beh.min

model2: AR ~ PC.Reporgan + PC2Beh.min

model3: AR ~ PC.Reporgan + PC1Beh.min + PC2Beh.min

df AICc

model0 6 1275.421

model1 7 1271.220

model2 7 1277.367

model3 8 1272.814

anova(model1,model3) Chisq = 0.434, P = 0.510

report model3

Random effects:

Groups Name Variance Std.Dev.

FID (Intercept) 1.2740 1.1287

MID:LINE (Intercept) 0.9653 0.9825

BLOCK (Intercept) 1.1790 1.0858

LINE (Intercept) 0.4150 0.6442

Number of obs: 1136, groups: FID, 238; MID:LINE, 177; BLOCK, 24; LINE, 12

Fixed effects:

Estimate Std. Error z value Pr(>|z|)

(Intercept) 1.11260 0.33156 3.356 0.000792 ***

PC.Reporgan -0.25344 0.14194 -1.786 0.074176 .

PC1Beh.min -0.17397 0.06981 -2.492 0.012699 *

PC2Beh.min -0.05306 0.08015 -0.662 0.507931

Summary: Adding PC.Reporgan as a covariate does not change the effect of PC1Beh.min or the lack of an effect of PC2Beh.min.

**GENETIC VARIATION IN MALES (AMONG INBRED LINES)**

(Note: that male only data is examined to model male eyespan and reproductive organ size, whereas the full data set with male-female pairs is required to model behaviour)

**1. Trait differences**

**Male.ES**

model0: Male.ES ~ (1 | BLOCK)

model1: Male.ES ~ (1 | LINE) + (1 | BLOCK)

Chisq = 9.8828 P = 0.001668 **

Random effects:

Groups Name Variance Std.Dev.

BLOCK (Intercept) 0.0008675 0.02945

LINE (Intercept) 0.0075011 0.08661

Residual 0.0588977 0.24269

Number of obs: 220, groups: BLOCK, 24; LINE, 12

Fixed effects:

Estimate Std. Error t value

(Intercept) 7.54935 0.03052 247.3

**Reproductive Organ Size**

model0: PC.Reporgan ~ (1 | BLOCK)

model1: PC.Reporgan ~ (1 | LINE) + (1 | BLOCK)

Chisq = 47.595 P = 5.24e-12 ***

Random effects:

Groups Name Variance Std.Dev.

BLOCK (Intercept) 0.00728 0.08533

LINE (Intercept) 0.54064 0.73528

Residual 0.89796 0.94761

Number of obs: 183, groups: BLOCK, 24; LINE, 12

Fixed effects:

Estimate Std. Error t value

(Intercept) -0.01697 0.22468 -0.076

**Behaviour**

**PC1Beh.min**

model0: PC1Beh.min ~ Female.ES + (1 | MID) + (1 | FID) + (1 | BLOCK)

model1: PC1Beh.min ~ Female.ES + (1 | LINE/MID) + (1 | FID) + (1 |

BLOCK)

Chisq = 5.8656 P = 0.01544 *

Random effects:

Groups Name Variance Std.Dev.

FID (Intercept) 0.05630 0.2373

MID:LINE (Intercept) 0.02256 0.1502

BLOCK (Intercept) 0.06094 0.2469

LINE (Intercept) 0.01147 0.1071

Residual 1.11807 1.0574

Number of obs: 2186, groups: FID, 238; MID:LINE, 224; BLOCK, 24; LINE, 12

Fixed effects:

Estimate Std. Error df t value Pr(>|t|)

(Intercept) -0.82529 0.44330 240.39000 -1.862 0.0639 .

Female.ES 0.14317 0.07635 242.82000 1.875 0.0620 .

**PC2Beh.min**

model0: PC2Beh.min ~ Female.ES + (1 | MID) + (1 | FID) + (1 | BLOCK)

model1: PC2Beh.min ~ Female.ES + (1 | LINE/MID) + (1 | FID) + (1 |

BLOCK)

Chisq = 4.2947 P = 0.03823 *

Random effects:

Groups Name Variance Std.Dev.

FID (Intercept) 3.257e-02 1.805e-01

MID:LINE (Intercept) 0.000e+00 0.000e+00

BLOCK (Intercept) 1.877e-15 4.333e-08

LINE (Intercept) 6.404e-03 8.002e-02

Residual 9.123e-01 9.552e-01

Number of obs: 2186, groups: FID, 238; MID:LINE, 224; BLOCK, 24; LINE, 12

Fixed effects:

Estimate Std. Error df t value Pr(>|t|)

(Intercept) 0.14281 0.31040 259.79000 0.460 0.646

Female.ES -0.02460 0.05373 257.31000 -0.458 0.647

**2. Outcomes**

**a) Attempts (BOBYQA)**

**covariates: Female.ES + PC1Beh.min + PC2Beh.min covariates**

model1: AT ~ Female.ES + PC1Beh.min + PC2Beh.min + (1 | MID) + (1 | FID) +

(1 | BLOCK)

model2: AT ~ Female.ES + PC1Beh.min + PC2Beh.min + (1 | LINE/MID) +

(1 | FID) + (1 | BLOCK)

Chisq = 7.4512, P = 0.00634 **

Random effects:

Groups Name Variance Std.Dev.

FID (Intercept) 0.2224 0.4716

MID:LINE (Intercept) 0.9222 0.9603

BLOCK (Intercept) 0.1088 0.3299

LINE (Intercept) 0.1545 0.3931

Number of obs: 2186, groups: FID, 238; MID:LINE, 224; BLOCK, 24; LINE, 12

Fixed effects:

Estimate Std. Error z value Pr(>|z|)

(Intercept) -1.8507 0.9868 -1.876 0.06072 .

Female.ES 0.5231 0.1695 3.086 0.00203 **

PC1Beh.min 2.0431 0.1996 10.235 < 2e-16 ***

PC2Beh.min 1.3272 0.2000 6.634 3.26e-11 ***

**b) Accepts**

**covariates: PC.Reporgan + PC1Beh.min (no Female.ES)**

model1: AR ~ PC.Reporgan + PC1Beh.min + (1 | MID) + (1 | FID) + (1 |

BLOCK)

model2: AR ~ PC.Reporgan + PC1Beh.min + (1 | LINE/MID) + (1 | FID) +

(1 | BLOCK)

Chisq = 9.7943, P = 0.001751 **

Random effects:

Groups Name Variance Std.Dev.

FID (Intercept) 1.2642 1.1244

MID:LINE (Intercept) 0.9654 0.9826

BLOCK (Intercept) 1.1676 1.0806

LINE (Intercept) 0.4086 0.6392

Number of obs: 1136, groups: FID, 238; MID:LINE, 177; BLOCK, 24; LINE, 12

Fixed effects:

Estimate Std. Error z value Pr(>|z|)

(Intercept) 1.10690 0.32973 3.357 0.000788 ***

PC.Reporgan -0.25443 0.14187 -1.793 0.072898 .

PC1Beh.min -0.16798 0.07075 -2.374 0.017587 *

**GENETIC VARIATION IN MALES (INBREEDING STATUS)**

(Note: that male only data is examined to model male eyespan and reproductive organ size, whereas the full data set with male-female pairs is required to model behaviour. t-tests use Satterthwaite approximations to degrees of freedom in 'lmerMod')

**1. Trait differences**

(Note: negative in.out values mean that inbred males had lower trait values than outbred males)

**Male.ES**

model0: Male.ES ~ (1 | LINE) + (1 | BLOCK)

model1: Male.ES ~ in.out + (1 | LINE) + (1 | BLOCK)

Chisq = 0.7564 P = 0.3845

Random effects:

Groups Name Variance Std.Dev.

BLOCK (Intercept) 0.0001598 0.01264

LINE (Intercept) 0.0074054 0.08605

Residual 0.0614842 0.24796

Number of obs: 236, groups: BLOCK, 24; LINE, 13

Fixed effects:

Estimate Std. Error df t value Pr(>|t|)

(Intercept) 7.54892 0.03010 10.81300 250.809 <2e-16 ***

in.out -0.09008 0.11023 11.85300 -0.817 0.43

**Reproductive Organ Size**

model0: PC.Reporgan ~ (1 | LINE) + (1 | BLOCK)

model1: PC.Reporgan ~ in.out + (1 | LINE) + (1 | BLOCK)

Chisq = 0.4077 P = 0.5231

Random effects:

Groups Name Variance Std.Dev.

BLOCK (Intercept) 0.03361 0.1833

LINE (Intercept) 0.55375 0.7441

Residual 0.87583 0.9359

Number of obs: 196, groups: BLOCK, 24; LINE, 13

Fixed effects:

Estimate Std. Error df t value Pr(>|t|)

(Intercept) -0.0200 0.2293 11.0550 -0.087 0.932

in.outout 0.4879 0.8205 10.8920 0.595 0.564

**Behaviour**

**PC1Beh.min**

model0: PC1Beh.min ~ Female.ES + (1 | LINE/MID) + (1 | FID) + (1 |

BLOCK)

model1: PC1Beh.min ~ in.out + Female.ES + (1 | LINE/MID) + (1 | FID) +

(1 | BLOCK)

Chisq = 0.0039 P = 0.9502

Random effects:

Groups Name Variance Std.Dev.

MID:LINE (Intercept) 0.02281 0.1510

FID (Intercept) 0.05170 0.2274

BLOCK (Intercept) 0.05859 0.2420

LINE (Intercept) 0.01150 0.1073

Residual 1.07666 1.0376

Number of obs: 2343, groups: MID:LINE, 240; FID, 238; BLOCK, 24; LINE, 13

Fixed effects:

Estimate Std. Error df t value Pr(>|t|)

(Intercept) -0.807476 0.420877 231.450000 -1.919 0.0563 .

Female.ES 0.140040 0.072424 232.720000 1.934 0.0544 .

in.outout 0.008838 0.147088 12.410000 0.060 0.9530

**PC2Beh.min**

model0: PC2Beh.min ~ Female.ES + (1 | LINE/MID) + (1 | FID) + (1 |

BLOCK)

model1: PC2Beh.min ~ in.out + Female.ES + (1 | LINE/MID) + (1 | FID) +

(1 | BLOCK)

Chisq = 0.9165 P = 0.3384

Random effects:

Groups Name Variance Std.Dev.

MID:LINE (Intercept) 4.583e-17 6.770e-09

FID (Intercept) 3.102e-02 1.761e-01

BLOCK (Intercept) 0.000e+00 0.000e+00

LINE (Intercept) 6.597e-03 8.122e-02

Residual 8.747e-01 9.353e-01

Number of obs: 2343, groups: MID:LINE, 240; FID, 238; BLOCK, 24; LINE, 13

Fixed effects:

Estimate Std. Error df t value Pr(>|t|)

(Intercept) 0.1451 0.2943 243.3200 0.493 0.622

Female.ES -0.0250 0.0509 240.5000 -0.491 0.624

in.out 0.1039 0.1149 12.4800 0.905 0.383

**2. Outcomes**

(Note: negative in.out values mean that inbred males made fewer mating attempts or were accepted less frequently than outbred males)

**a) Attempts (BOBYQA)**

**covariates: Female.ES + PC1Beh.min + PC2Beh.min covariates**

model1: AT ~ Female.ES + PC1Beh.min + PC2Beh.min + (1 | LINE/MID) +

(1 | FID) + (1 | BLOCK)

model2: AT ~ in.out + Female.ES + PC1Beh.min + PC2Beh.min + (1 |

LINE/MID) + (1 | FID) + (1 | BLOCK)

Chisq = 0.065, P = 0.7987

Random effects:

Groups Name Variance Std.Dev.

MID:LINE (Intercept) 1.09207 1.0450

FID (Intercept) 0.19222 0.4384

BLOCK (Intercept) 0.09213 0.3035

LINE (Intercept) 0.12814 0.3580

Number of obs: 2343, groups: MID:LINE, 240; FID, 238; BLOCK, 24; LINE, 13

Fixed effects:

Estimate Std. Error z value Pr(>|z|)

(Intercept) -1.9080 0.9592 -1.989 0.04668 *

Female.ES 0.5309 0.1649 3.220 0.00128 **

PC1Beh.min 1.9992 0.1908 10.478 < 2e-16 ***

PC2Beh.min 1.2565 0.1942 6.471 9.73e-11 ***

in.out 0.1308 0.5128 0.255 0.79859

**b) Accepts**

**covariates: PC.Reporgan + PC1Beh.min (no Female.ES)**

model1: AR ~ PC.Reporgan + PC1Beh.min + (1 | LINE/MID) + (1 | FID) +

(1 | BLOCK)

model2: AR ~ PC.Reporgan + PC1Beh.min + in.out + (1 | LINE/MID) +

(1 | FID) + (1 | BLOCK)

Chisq = 0.7548, P = 0.385

Random effects:

Groups Name Variance Std.Dev.

FID (Intercept) 1.3510 1.1623

MID:LINE (Intercept) 1.3112 1.1451

BLOCK (Intercept) 1.0133 1.0066

LINE (Intercept) 0.2961 0.5442

Number of obs: 1222, groups: FID, 238; MID:LINE, 190; BLOCK, 24; LINE, 13

Fixed effects:

Estimate Std. Error z value Pr(>|z|)

(Intercept) 1.13953 0.30903 3.687 0.000227 ***

PC.Reporgan -0.35260 0.14529 -2.427 0.015229 *

PC1Beh.min -0.17323 0.07078 -2.447 0.014387 *

in.out -0.65377 0.74652 -0.876 0.381164
